# Assist and resist: a dual-track framework for pediatric exoskeleton rehabilitation

**DOI:** 10.3389/fped.2026.1911727

**Published:** 2026-08-31

**Authors:** Hua Xia, Xia Liu, Bo Liu, Yang Zhang

**Affiliations:** 1 College of Physical Education, Hunan Normal University, Changsha, China; 2 Independent Researcher, Windermere, FL, United States

**Keywords:** exoskeleton, muscular abnormality, rehabilitation, resistance training, strength training

## 1 Introduction

Wearable exoskeletons have usually entered pediatric rehabilitation through a familiar promise: to assist movement that disease, weakness, or impaired motor control has made difficult. This logic is clinically intuitive. For children with neuromotor disorders, robotic assistance may help them stand, step, practice gait, or experience movements otherwise unavailable to them. Yet recent work using a lightweight portable isokinetic resistance robot in children with spinal muscular atrophy (SMA) (1) raises a different question: should the next generation of pediatric exoskeletons not only assist movement, but also safely resist it?

This question is especially relevant because pediatric rehabilitation occurs during growth. Children with neuromuscular disease, cerebral palsy (CP), developmental delay, and related neuromotor disorders are not simply smaller adults undergoing functional recovery. Their musculoskeletal systems, motor strategies, neural pathways, and participation habits are developing while disease-related weakness, disuse, spasticity, fatigue, or impaired motor control may constrain future functional reserve. The clinical objective is therefore not only to improve movement today, but also to preserve or redirect developmental trajectories.

In six children with SMA type II, a six-week program using a portable isokinetic knee-training robot produced improvements across functional, muscular, and neurophysiological outcomes (1). These preliminary findings cannot establish efficacy across pediatric neuromotor disorders. They nevertheless introduce an important therapeutic proposition: pediatric exoskeleton rehabilitation need not be restricted to assistance toward easier or more normative movement. In selected children, appropriately dosed resistance may provide an additional stimulus for muscular and neuromuscular adaptation.

## 2 From assisting movement to training adaptation

Most lower-limb exoskeletons in research and development are designed primarily to reduce effort or facilitate movement. This may be appropriate when the immediate goals are mobility, energy conservation, access to standing and walking, or repeated task-specific practice. Wearable lower-limb exoskeletons have increasingly been explored for gait training and mobility support across neuromuscular impairments (2). In pediatric SMA, experience with the ATLAS 2030 gait exoskeleton has suggested potential improvements in selected strength and range-of-motion outcomes (3), although its therapeutic logic remains predominantly assistive. Assistance, however, does not necessarily provide the same physiological stimulus as training designed specifically to increase force production. When the therapeutic target includes muscle strength, muscle morphology, motor-unit recruitment, or functional carryover after device removal, the ability to prescribe and progressively adjust effort may become important.

The portable robot evaluated in SMA type II weighed 0.96 kg and provided isokinetic resistance during knee extension (1). Isokinetic resistance is conceptually attractive in children with substantial weakness because movement velocity can be controlled while resistance responds to the user's force output. Compared with fixed external loading, such control may allow meaningful effort across the available range of motion while reducing abrupt or excessive loading. Isokinetic training has previously been used to improve strength and balance in other pediatric disability populations, although the underlying conditions and training contexts differ substantially (4).

After six weeks of high-intensity training, improvements were observed in sit-to-stand performance, peak knee-extension torque, range of motion, work per extension, quadriceps cross-sectional and volumetric measures, physiological cross-sectional area, and femoral nerve compound muscle action potential (1). The convergence of functional, muscular, and neurophysiological changes provides signals consistent with training-induced neuromuscular adaptation. Nevertheless, the small uncontrolled study cannot determine how much of the observed change was attributable specifically to robotic resistance training or whether these gains persist after training ends. The findings should therefore be viewed as preliminary proof of therapeutic potential rather than evidence of established efficacy.

## 3 Why the pediatric growth window changes the argument

The pediatric context changes the significance of resistance training. In adults, resistance exercise is commonly discussed as a means of restoring or preserving function. In children with disabilities, it may also contribute to the developmental accumulation of strength, muscle size, joint control, and confidence in physical tasks (5). Even modest improvements during growth may expand the activities a child is able to practice, potentially influencing subsequent motor learning and participation.

Evidence from CP illustrates why muscle development deserves particular attention. Resistance training has shown favorable effects on proxy indicators of muscle fiber diameter in ambulatory children with CP, although evidence for power training has been less consistent for hypertrophy-related outcomes (6). Importantly, much of this evidence comes from ambulatory children with lower levels of disability, leaving uncertainty about how an adequate strengthening stimulus can be delivered to children with greater weakness or functional limitation.

Portable resistive robotics could help address this therapeutic gap by allowing training within a controlled range: sufficiently demanding to generate muscular effort, yet sufficiently constrained to reduce excessive loading, compensatory movement, or fatigue. This may be particularly relevant when disease-related weakness or disuse interferes with the normal accumulation of muscle and strength during growth. In SMA, natural-history studies have documented limited or declining motor trajectories in affected children (7, 8). In CP, altered muscle morphology and reduced muscle volume contribute to weakness, impaired joint power, gait limitations, and longer-term musculoskeletal consequences (9, 10).

Growth itself must also be considered when interpreting intervention-related morphological change. Muscle morphology and strength change measurably during normal childhood development (11), whereas thigh muscle volume in children with SMA may remain relatively stable over comparable periods (12). Against this background, the short time course of the quadriceps changes observed after six weeks of robot-mediated training is noteworthy, but controlled studies are needed before those changes can be attributed specifically to the intervention. The growth window is therefore not merely a background characteristic of pediatric rehabilitation. It is part of the therapeutic context in which training dose, movement opportunities, and physiological adaptation may interact over time.

## 4 Beyond SMA: promise, but not one-size-fits-all

The most immediate relevance of the available resistive robotic evidence is to SMA type II. Pharmacological advances have altered survival and disease trajectories in SMA, but they do not fully normalize neuromuscular or functional development (13, 14). Exercise and strengthening remain potentially important adjuncts, although existing trials have produced mixed findings and have highlighted practical difficulties in achieving sufficient training intensity and adherence (15, 16). Neuromodulatory approaches have further broadened the therapeutic landscape, including recent work on non-invasive and epidural spinal cord stimulation (17, 18). Collectively, these developments reinforce the likelihood that future rehabilitation will combine biological, technological, and activity-based approaches rather than rely on any single modality.

The broader implication of resistive exoskeleton technology is therefore not that one SMA-derived protocol should be transferred across pediatric diagnoses. Rather, the platform provides a means of testing diagnosis-specific questions. Potential applications might include selected children with SMA type II or III, CP with marked antigravity weakness, developmental disorders associated with reduced lower-limb strength, selected congenital neuromuscular conditions, or severe deconditioning after prolonged immobilization. The therapeutic objective would differ across these populations—from preserving force-generating capacity to improving muscle morphology, joint power, antigravity strength, or functional reserve.

Such extension requires caution. Progressive neuromuscular disorders differ in their responses to mechanical loading. In muscular dystrophies and some myopathies, excessive resistance, eccentric stress, fatigue accumulation, and muscle damage are particular concerns. In children with spasticity or dystonia, inappropriate resistance could reinforce compensatory movement strategies or increase discomfort. Contractures, skeletal fragility, scoliosis, cardiopulmonary impairment, and severe fatigue may introduce further limitations. The appropriate conclusion is therefore not broad adoption, but diagnosis-specific evaluation. The therapeutic dose, movement mode, developmental stage, and safety phenotype must all be considered before resistance can be incorporated into pediatric exoskeleton rehabilitation.

## 5 Portability and home rehabilitation

Portability may substantially influence the clinical value of pediatric rehabilitation technologies because achievable training dose is often constrained by transportation, clinic availability, caregiver time, cost, and child engagement (19, 20). A home-compatible system could shift strengthening from episodic clinic-based treatment toward more frequent training integrated into everyday life.

Several features are particularly relevant. Adjustable resistance could allow progression as strength changes, avoiding the problem of a fixed exercise being too demanding initially or insufficient after adaptation. Embedded sensing could quantify torque, joint angle, velocity, range of motion, adherence, and fatigue-related decline, making treatment dose more measurable. Interactive or gamified feedback could also help sustain engagement during repetitive training.

Home use, however, transfers part of the responsibility for rehabilitation from clinicians to families. Caregivers may effectively become co-therapists, creating challenges related to supervision, training burden, recognition of adverse events, and access to clinical support. Digital literacy, family resources, and device affordability may also determine who can benefit from home robotic rehabilitation.

Future studies should therefore evaluate implementation alongside physiological and functional efficacy: who can use the device safely, how much caregiver training is required, what remote monitoring is necessary, and whether increased access to training can be achieved without increasing burden or inequity.

## 6 Discussion

The initial findings from portable resistive robotic training in SMA type II are encouraging but should be interpreted within a broader pediatric rehabilitation literature. The study involved only six participants, lacked a randomized control group, and evaluated a prototype centered on concentric isokinetic knee extension (1). Its value therefore lies less in establishing clinical efficacy than in demonstrating that precisely controlled wearable resistance is technically feasible and may produce measurable functional, muscular, and neurophysiological responses in children with severe weakness.

Existing evidence also cautions against assuming that resistance itself is sufficient to generate meaningful functional change. Conventional resistance training in children with SMA has demonstrated feasibility and some favorable outcomes, but randomized exercise studies have also produced limited or negative results (15, 16). Similarly, resistance-based interventions in CP may improve aspects of muscle morphology, yet the available evidence is concentrated in more ambulatory children and does not establish that greater muscle adaptation will necessarily translate into improved activity or participation (6). The potential advantage of robotic resistance may therefore lie not simply in adding load, but in improving how that load is controlled, individualized, progressed, and measured. Whether these technological advantages translate into superior or more durable clinical outcomes remains unknown.

The comparison with assistive exoskeletons is equally important. Assistance should not be viewed as therapeutically inferior to resistance. For a child who cannot independently generate sufficient movement, assistance may enable task-specific repetition and upright mobility while creating opportunities for motor learning and participation (2, 3). Resistance, in contrast, may be particularly useful when the principal objective is to increase active force production or provide a progressive training stimulus. It can also carry disadvantages: excessive resistance may increase fatigue, encourage compensatory strategies, or impose inappropriate mechanical stress in susceptible children.

The emerging question is therefore not whether pediatric exoskeletons should assist or resist, but how these modes should be selected, sequenced, and potentially combined. A child might initially require assistance to access a movement, progress toward reduced assistance as motor capacity improves, and subsequently tolerate controlled resistance to increase training demand. Other children may continue to benefit primarily from assistance, whereas some may be suitable for resistance from the outset. The appropriate balance is likely to depend on diagnosis, functional capacity, developmental stage, treatment goals, fatigue, and safety.

Several priorities follow. Larger multicenter studies will be particularly important in rare pediatric disorders where individual centers cannot readily recruit sufficient samples. Depending on the clinical context, designs could include randomized parallel-group trials, delayed-start controls, or adaptive protocols. Outcomes should extend beyond short-term torque and task performance to include growth-adjusted muscle morphology, motor milestones, participation, fatigue, pain, falls, caregiver burden, and retention after device withdrawal. Dosing studies are equally important. The effects of resistance intensity, movement velocity, session frequency, repetition number, stiffness, and recovery intervals are unlikely to be uniform across ages and diagnoses. Longitudinal safety should also be treated as more than the absence of acute adverse events. Pediatric technologies should be evaluated for whether repeated use contributes to pain, excessive fatigue, maladaptive compensation, fear, caregiver burden, or inequitable access over months and years.

**Figure 1:**
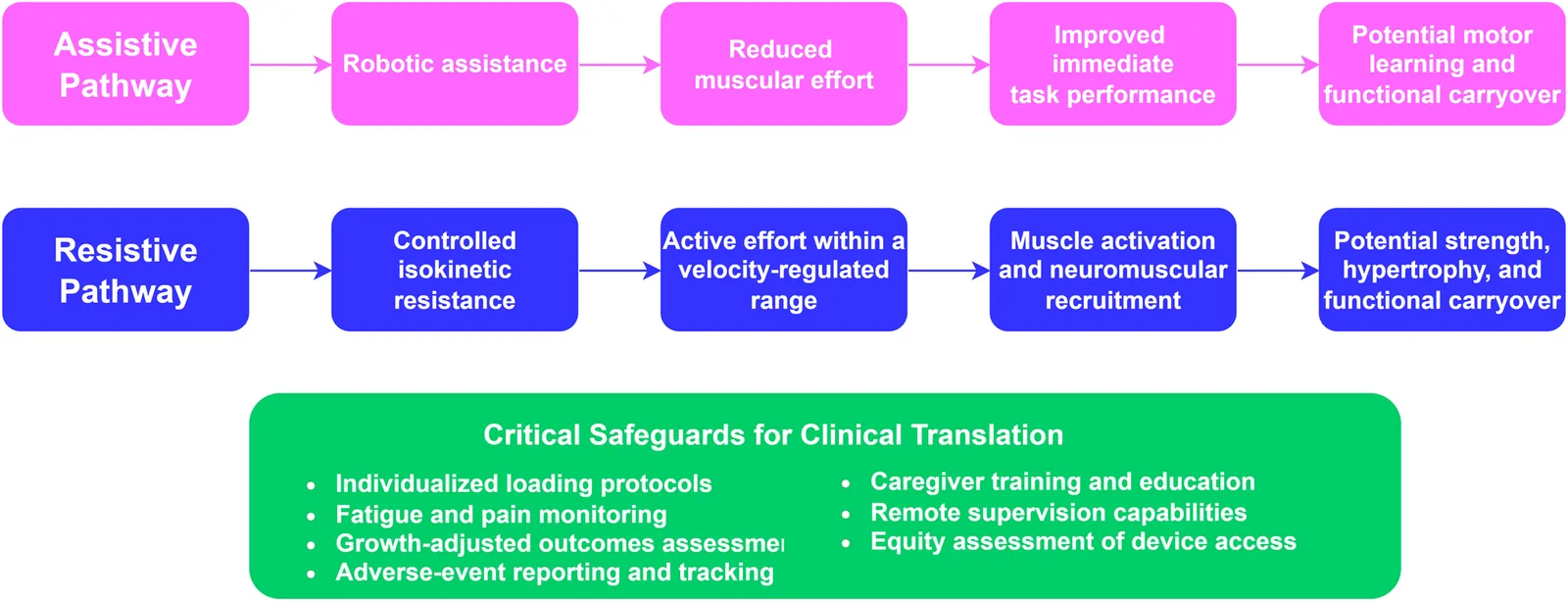
Assist and resist: a dual-track framework for pediatric exoskeleton rehabilitation.

The complementary roles of assistive and resistive exoskeleton modes, together with the developmental and clinical safeguards required for pediatric translation, are summarized in Figure 1. Pediatric wearable robotics should therefore progress from engineering feasibility toward developmentally informed rehabilitation science. The central task is not to identify a universally superior mode, but to determine how assistance and resistance can be matched to therapeutic objectives and safely adjusted as the child develops.

## 7 Conclusion

Pediatric exoskeleton rehabilitation should not be framed as a simple choice between assistance and resistance. Assistance can provide access to movement, repetition, and participation, whereas appropriately dosed resistance may provide an additional stimulus for muscular and neuromuscular adaptation. The early findings from portable resistive robotic training in SMA type II provide important preliminary proof-of-concept evidence, but not yet evidence for broad clinical adoption. Future research should determine when, for whom, and at what dose assistive and resistive modes should be used, sequenced, or combined. An individualized dual-track framework—grounded in diagnosis, developmental stage, therapeutic goals, and long-term safety—may offer a more productive direction for pediatric exoskeleton rehabilitation.
